# Dodecanol, metabolite of entomopathogenic fungus *Conidiobolus coronatus*, affects fatty acid composition and cellular immunity of *Galleria mellonella* and *Calliphora vicina*

**DOI:** 10.1038/s41598-021-95440-6

**Published:** 2021-08-05

**Authors:** Michalina Kazek, Agata Kaczmarek, Anna Katarzyna Wrońska, Mieczysława Irena Boguś

**Affiliations:** 1 The Witold Stefański Institute of Parasitology, Polish Academy of Sciences, ul. Twarda 51/55, 00-818 Warsaw, Poland; 2 BIOMIBO, ul. Strzygłowska 15, 04-872 Warsaw, Poland

**Keywords:** Fatty acids, Entomology, Fungal host response, Fungal immune evasion, Fungal pathogenesis, Immune cell death, Apoptosis

## Abstract

One group of promising pest control agents are the entomopathogenic fungi; one such example is *Conidiobolus coronatus*, which produces a range of metabolites. Our present findings reveal for the first time that *C. coronatus* also produces dodecanol, a compound widely used to make surfactants and pharmaceuticals, and enhance flavors in food. The main aim of the study was to determine the influence of dodecanol on insect defense systems, i.e. cuticular lipid composition and the condition of insect immunocompetent cells; hence, its effect was examined in detail on two species differing in susceptibility to fungal infection: *Galleria mellonella* and *Calliphora vicina*. Dodecanol treatment elicited significant quantitative and qualitative differences in cuticular free fatty acid (FFA) profiles between the species, based on gas chromatography analysis with mass spectrometry (GC/MS), and had a negative effect on *G. mellonella* and *C. vicina* hemocytes and a Sf9 cell line in vitro: after 48 h, almost all the cells were completely disintegrated. The metabolite had a negative effect on the insect defense system, suggesting that it could play an important role during *C. coronatus* infection. Its high insecticidal activity and lack of toxicity towards vertebrates suggest it could be an effective insecticide.

## Introduction

In their natural environment, insects have to cope with a variety of microorganisms, and as such, have developed a complex and efficient defense system. The first line of defense is a cuticle formed of several layers, with an epicuticle on the outside, a procuticle underneath it and an epidermis beneath that. The epicuticular layer is covered by a wax barrier against water. About 70% of the cuticle is composed of proteins, with the remaining 30% being made up of chitin, lipids, chinons and phenols. The cuticle is covered with complex mixtures of nonpolar and polar compounds^1^, including various hydrocarbons, free fatty acids (FFAs), alcohols, waxes, glycerides, aldehydes and sterols. The FFA profile can differ between insect groups and even between developmental stages of the same species. In addition, some specific cuticular lipids may also have a role in defending against fungal invasion^2–4^, and the presence of antagonistic microbes, toxic lipids and cuticle phenolic compounds has been suggested to inhibit fungal growth^4–7^. Cuticular fatty acids are known to have a profound effect on fungal growth and spore germination; for example, *C. vicina* larvae and pupae demonstrate remarkable resistance to mycosis, which has been attributed to fatty acid composition of their epicuticle^3,8,9^.

Insects employ multiple immune responses incorporating both humoral and cellular defense reactions. The humoral reactions include the production of antimicrobial peptides, reactive oxygen and nitrogen species, and the activation of the prophenoloxidase system, which regulates the coagulation and melanization of hemolymph. Cellular responses, on the other hand, are based around the phagocytosis of small pathogens such as bacteria and fungi, the encapsulation of pathogens such as parasitoids and nematodes, and nodulation by specific insect immune cells (hemocytes)^10,11^.

A variety of methods have been developed to control insect populations. The most common approach is based on the use of cheap, quick-acting, and easily-accessible chemical insecticides. However, as chemical pesticides can also have a negative impact on the environment, there is a need to identify more specific agents, and the search has naturally turned to the natural microbial enemies of insects. Many naturally-occurring viral, bacterial or fungal entomopathogens are considered to play a vital role as biological control agents for insect populations in the wild, and several species of entomopathogenic fungi are currently employed as biological control agents for insect pests^12,13^.

The fungal infection process starts with the adhesion of blastospores or conidia to the insect body. The success of this step depends on the hydrophobic and electrostatic forces present on the cuticle^14^, as well as the nature of the enzyme cocktail, a mixture of lipases, proteases and chitinases, which is secreted by fungi to hydrolyze the insect epidermis. In addition, fungi produce specialized structures including penetration pegs and/or appresoria, which are used to penetrate the host integument. As cuticle composition strongly affects the germination of conidia, different insects show differential susceptibility to pathogenic fungi^15^. Following attachment, the insect haemocoel is penetrated by fungal hyphae, which grow into the internal organs^16,17^. In addition, some fungi are known to produce mycotoxins, such as small secondary metabolites, cyclic peptides and macromolecular proteins, which can cause paralysis or disrupt physiological processes, including the immune responses of the insect^16,18,19^. Successful penetration of the host body is followed by the death of the host, either by mechanical damage of the internal organs, the action of toxic metabolites or exhaustion of nutrients. Due to their ability to infect and kill insects, entomopathogenic fungi offer great potential as biological control agents. Amongst the currently-known entomopathogenic fungi, the Entomophthorales such as *Furia, Conidiobolus, Erynia* or *Entomophaga*, demonstrate the highest insecticidal activity; however, *Beauaveria, Hirsutella, Isaria, Metarhizium* and *Paecilomyces* are presently used as biopesticides^16,20–22^.

Entomopathogenic fungi also produce insecticidal metabolites, and these can represent attractive alternatives to chemical insecticides. One evolutionarily primitive member of the Entomophthorales, the soil fungus *C. coronatus* (genus *Conidiobolus*) demonstrates entomopathogenicity towards various insects, including *Dendrolimus pini, Chortippus brunei, Stenobothrys lineatus* and *G. mellonella*^23^, as well as various collembolans^24^. It is also known to produce mycotoxins which affect the insect immune system^19,25^. Our previous research showed that *C. coronatus* produces two β- carboline alkaloids, harman and norharman, both of which delay pupation and adult eclosion, and inhibit total monoamine oxidase activity in *G. mellonella*. In addition, they increase the serotonin concentration and decrease the monoamine oxidase A level in the heads of moths, and stimulate the phagocytic activity of hemocytes^26,27^. *C. coronatus* also produces two key insecticidal proteins: cronatin-1 (36 kDa), showing both elastolytic and chitinolytic activities, and coronatin-2 (14.5 kDa), which does not appear to demonstrate any enzymatic activity. Both proteins are toxic to *G. mellonella* hemocytes, being known to disintegrate the nets formed by granulocytes and plasmatocytes by rapid degranulation of granulocytes, and to cause extensive vacuolization of plasmatocytes accompanied by cytoplasm expulsion and cell disintegration^19,25^.

Our present work demonstrates that *C. coronatus* releases dodecanol into its culture media. Not only does Dodecanol appear to be an effective potential insecticide, and a metabolite of C*. coronatus*, it is also known to be safe towards vertebrates, being currently approved for use as a food additive. However, it is essential to have a clear understanding of the complex nature of the interactions between hosts and pathogens to create innovative and effective biocontrol agents.

The main aim of this work was to determine the effects of dodecanol, a metabolite of *C. coronatus,* on insect defense systems in two insect models differing in their susceptibility to *C. coronatus* infection: the susceptible *G. mellonella* (Lepidoptera) and the resistant *C. vicina* (Diptera). It is hypothesised that the application of dodecanol might affect the FFA cuticular profiles and/or cellular immune system of the host, thus weakening its defence. Such a result would indicate that dodecanol plays an important role in the development of mycosis.

## Methods

### Insects

All insects used in experiments were reared in the laboratory under optimal growth conditions.

The wax moth *G. mellonella* (Pyralidae, Lepidoptera), was reared in glass chambers at 30 °C, 70% relative humidity and in constant darkness. The insects were maintained on a semi-artificial diet as described by Sehnal^28^. Five-day-old last instar larvae (wandering stage) which had ceased feeding and mature six-day-old adults were used for experiments.

The blow fly *C. vicina* (Calliphoridae, Diptera) specimens were reared at 25 °C, 70% relative humidity and a long-day photoperiod (L:D 16:8). Adults were kept in the glass tank with unlimited access to water supplemented with glucose and beef meat. The larvae were fed on beef ad libitum. The insects were maintained in the same conditions up to the sixth generation. The last third instar larvae (wandering stage) and six-day-old mature adults were used in the experiments.

### Fungus

*C. coronatus* strain number 3491, isolated from *Dendrolaelaps spp*, was used for infection. The fungus was received from the collection of Professor Błazy (Polish Academy of Science, Research Center for Agricultural and Forest Environment, Poznań). The fungal colonies were routinely cultured in 90 mm Petri dishes on Sabouraud agar (SAB) medium supplemented with homogenized *G. mellonella* larvae to a final concentration of 10% wet weight (SAB-GM) in order to increase virulence. They were incubated at 20 °C under a 12-h photoperiod (L:D 12:12) to stimulate sporulation^29^. The fungal SAB-GM colonies used for all experiments were cultured for seven days.

Sporulating SAB-GM colonies were rinsed with sterile water to harvest the conidia, and 100 μl portions of the suspension, containing approximately 52 conidia per each, were used for the inoculations of cultural media. The number of conidia was counted in a Bürker chamber under a light microscope. Next, *C. coronatus* was cultivated for four weeks at 20 °C in 500 ml Erlenmeyer flasks containing 250 ml of MM (0.1% (NH4)2SO4, 0.45% KH2PO4, 1.05% K2HPO4, 0.05% sodium citrate dehydrate, 0.2% glucose and 0.025% MgSO4) and LB (1% tryptone, 0.5% yeast extract, 0.5% NaCl and 0.1% 1 M NaOH) media, as described previously^30^. After incubation, the mycelia were removed by filtration through Whatman no.1 filter paper, and the cell-free filtrates were taken for SPE extraction and further GC/MS analysis.

### Fungal infection and dodecanol application

The insects were exposed for 24 h to fully-grown and sporulating *C. coronatus* colonies. Around 20 larvae of *G. mellonella* and *C. vicina* were maintained in each Petri dish containing the fungal colony; in addition, control groups were formed from larvae exposed for 24 h to sterile SAB-GM medium. This was found to be the most efficient method of infection, being one that most closely resembles the natural infection process^31^. Approximately 30 adult insects were placed in plastic boxes with open Petri dishes with or without fungus. After exposure, the insects were either immediately frozen or transferred to new, clean Petri dishes/new plastic boxes with appropriate food, and kept there at an appropriate temperature and lighting conditions for 24 h.

Dodecanol (Merck), identified as a one of metabolites of *C. coronatus*, was directly administered to the dorsal side of the insect cuticle by topical application. During the experiments on *C. vicina*, the substance was dissolved in 99.8% ethanol (AVANTOR); each insect was treated with 1 µl of the mixture containing 100 µg of substance. For *G. mellonella*, dodecanol was dissolved in acetone (AVANTOR), which provides better penetration of the wax moth cuticle^32^ and 5 µl solution at a concentration 100 µg/µl was applied to each insect. The doses were calculated and adjusted to the body weights of the insects and their sensitivity established in pilot studies; the mean weights of the *C. vicina* adults and larvae were 65 mg and 90 mg, while those of *G. mellonella* moths and larvae were 80 mg and 200 mg, respectively.

For all experiments, two control groups were used: untreated insects and insects treated with 1 µl ethanol 99.8% or 5 µl of acetone. After dodecanol application, the insects were reared with their optimal growth conditions as described above. The insects were collected 24 h after treatments and kept at -80 °C until analysis.

### Larval hemolymph collection and hemocyte cultures

The hemocyte cultures were established from freshly-collected hemolymph of *G. mellonella* and *C. vicina* larvae. The last instar larvae (wandering stage) were used in all experiments. Before bleeding, the larvae were washed with distilled water and then immersed briefly in 70% (v/v) ethanol to sterilize their surfaces. In the case of *G. mellonella*, larval hemolymph was collected from small incisions in the last proleg. One drop of hemolymph (26 µl) contained 1.3 × 10^5^ cells. The *C. vicina* larvae were punctured in the distal area using sterile entomological pins, then a drop of hemolymph was squeezed gently out. One drop of hemolymph (17 µl) contained 5 × 10^5^ cells. In both cases, freely-dripping hemolymph from insects was collected into sterile polypropylene 1.5 ml centrifuge tubes preloaded with Grace Insect Medium (GIM; Gibco) with added gentamycin (10 mg/ml; Gibco), amphotericin B (250 µg/ml; Gibco) and 0.1 mM phenylthiourea (PTU; Merck).

The fresh hemolymph (6–10 drops) was mixed with 300 μl of GIM. The hemolymph suspension was immediately transferred to 48-well culture plates with a glass bottom (Nest). Each plate well was filled with 100 µl of the hemocyte mix and supplemented to 400 µl with clean GIM; this was then incubated for 24 h at 27 °C and 80–90% humidity. Following this, 1 µl of ethanol containing 100 µg of dodecanol (final concentration 0.28 µg/µl) was added. In addition, 1 µl volumes of 99.8% ethanol and untreated cells were used as controls. Following this, the plates were incubated for another 24 or 48 h, depending on the group, at 27 °C and 80–90% humidity. Each culture was performed as five or six independent replicates.

The obtained hemocyte cultures were analyzed using an inverted AxioVert A1 fluorescence microscope with phase contrast (Zeiss), equipped with an Axio Cam ICc 5 camera (Zeiss) and Zen lite 2012 software (Zeiss), and/or an Olympus (type IX 50) inverted phase contrast microscope with Color View (IIIu) camera connected with Cell D software.

### Sf9 line and WST-1 test

The Sf9 cell line (Thermo Fisher Scientific) was derived from the pupal ovarian tissue of the fall army worm, *Spodoptera frugiperda* (Lepidoptera). The Sf9 cells were cultured in optimal growth conditions at 27° C in the appropriate TNM-FH medium (Merck). Passages were performed every five to seven days according to the manufacturer's recommendations.

To each well of a 48-well culture plate, 300 µl of medium and 50 µl of stock containing approximately 2.5–3 × 10^5^ cells were added. The cells were incubated for 24 h, then 1 µl of ethanol containing 100 µg of dodecanol (final concentration 0.28 µg/µl) was added. Following this, the cells were incubated for another 24 or 48 h, and changes in cell morphology were observed. Additionally, cell proliferation and viability were measured using the WST-1 test. Briefly, 30 µl of WST-1 substance (Roche) were added to each plate well, the absorbance was read at 440 and 650 nm after 3 h and the results were calculated according to the manufacturer’s instructions. Only the Sf9 cell line was tested after dodecanol treatment, as insect hemocytes do not proliferate in vitro. Each test was carried out in three independent replicates.

### Immunohistochemical visualization of apoptotic bodies

To characterize the effect of dodecanol on the internal organs of *G. mellonella* and *C. vicina*, 24 h after topical application, last instar larvae (wandering stage) were used. Untreated larvae and insects treated with acetone (*G. mellonella*) or ethanol (*C. vicina*) were used as controls. Before fixation, the larvae were washed with distilled water, then immersed briefly in 70% (v/v) ethanol to sterilize their surfaces. The larvae were immediately fixed overnight at 4ºC in a solution of 4% paraformaldehyde in 0.1 M Na-phosphate buffered saline (PBS), pH 7.4. After fixation, the larvae were washed three times in 0.1 M PBS and dehydrated in an increasing series of ethanol and chloroform, then embedded in paraffin wax (melting temperature 53–57 °C; Merck), cut into 8-μm-thick sections and mounted on SuperFrost Plus glass slides (Thermo Fisher Scientific).

Following paraffin removal and rehydration, the sections were stained to identify apoptotic cells by the TUNEL method with an In situ Apoptosis Detection Kit (Abcam). Briefly, terminal deoxynucleotidyl transferase (TdT) catalyzes the incorporation of deoxynucleotides at the free 3′-OH ends of fragmented DNA. The biotinylated nucleotides are bound with a streptavidin–horseradish peroxidase (HRP) conjugate, and diaminobenzidine (DAB) is reacted with the HRP to generate an insoluble brown substrate at the site of DNA fragmentation. Methyl green aids were used to differentiate normal and apoptotic cells. The prepared slices were mounted in HIGHDEF IHC fluoromount (Enzo Life Sciences) and inspected under microscope.

### Extraction of samples, derivatization and GC/MS analysis

To isolate the surface lipid components for GC/MS analysis, the larvae (20 individuals for each sample) were extracted for five minutes (130 RPM, room temperature) in 20 mL of dichloromethane (Merck). The extracts were then placed in glass flasks and evaporated under nitrogen.

The FFA extracts were treated with 100 μl of BSTFA:TMCS mixture 99:1 (Merck), and heated for 1 h at 100 °C to form trimethylsilyl esters (TMS). The TMSs of the fatty acids were then analyzed by GC/MS using a GCMS-QP2010 system with mass detector (Shimadzu) and NIST 11 library. As an internal standard (IS), the 19-methylarachidic acid (Merck; 1 mg/ml) was used, because it separates well from all the sample constituents and was not previously present in the insect samples^30^. The mass spectra of the tested trimethylsilyl esters revealed the presence of the following ions: M + (molecular ion), [M-15] + , and fragment ions at m/z 117, 129, 132. The contents were calculated by comparing the relative peak areas with the IS peak area. All constituents were assumed to have response factors of one. Helium was used as the carrier gas. The analysis was performed in split-injection mode using a ZB-5MSi (Zebron) column (thickness 0.25 µm, length 60 m, diameter 0.25 µm). The column oven temperature cycle was held at 80 °C for three minutes and then ramped from 80 to 310 °C at 4 °C /minute; the final temperature was then held for 10 min. The ion source temperature was 200 °C. The interface temperature was 310 °C. The method is also based on literature^33–35^.

The filtrates were subjected to solid phase extraction (SPE) SmartPrep (Horizon). Each filtrate (100 ml) was prepared for extraction in three independent replicates. Waters OASIS MCX 8 150 mg cartridges were conditioned with 5% ammonia water in methanol (90:10) and pure methanol. The loaded samples were eluted with 10 ml methanol (1 ml/min), then the extract was evaporated under nitrogen to dry weight, and the samples were used for GC/MS analysis.

The GC/MS analysis of the compounds released by *C. coronatus* into culture medium was carried out on the same GC/MS system under the same conditions as described above but with a DM-5 MS column (Zebron, Phenomenex, length 30.0 m, thickness 0.25 μm, diameter 0.25 mm). N-decane was used as an internal standard. The mass spectrum of the trimethylsilyl esters of alcohols revealed the presence of the following ions: M + (molecular ion), [M-15] + , and fragment ions at m/z 73, 75, 103. The analyses were performed in three independent replications for each sample (test and control).

### Statistics

The obtained results were tested using the Student’s t-test and ANOVA. Post hoc analysis was performed using Tukey’s test, with the results being significant at *p* ≤ 0.05. The normality of the data was tested using the Kolmogorov–Smirnov test. STATISTICA (StatSoft Polska) and Prism-GraphPad software were used for the analysis.

## Results

### Dodecanol production

**Figure 1:**
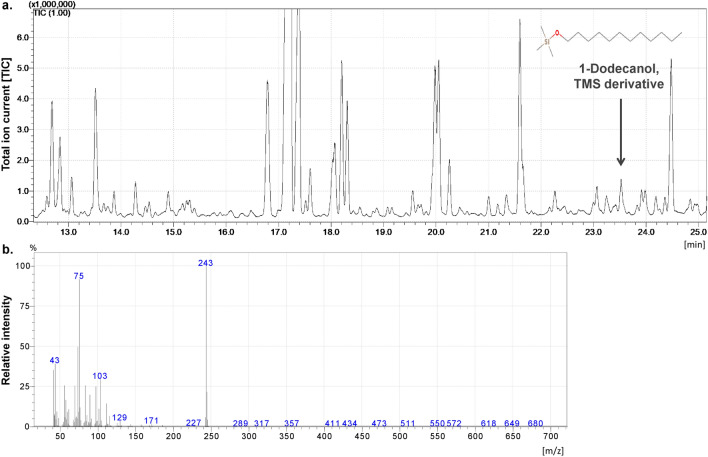
The total ion current (TIC) of substances in the four-week-old MM media where *Conidiobolus coronatus* were grown with dodecanol (TMS ester), pick marked (**a**) and mass spectrum of dodecanol (**b**).

GC/MS analysis of the minimal medium (MM) and rich medium (LB) where *C. coronatus* was cultured confirmed the presence of dodecanol (M + m/z = 243; retention time 23.60 min) (Fig. 1). The cell-free fungal filtrates were analyzed by GC–MS as described in Materials and Methods. Sterile MM and LB media were used as controls. The quantitative profiles of the dodecanol produced by *C*. *coronatus* were found to depend on the type of culture medium: *C. coronatus* cultured in MM medium produced significantly more dodecanol than that propagated in rich LB medium—twice the amount was observed during the three-week incubation (Student’s t-test *p* < 0.001) and 1.4 times the amount during the four-week incubation (Student’s t-test *p* = 0.001) (Supplementary Table 1).

### Effect of fungal infection and dodecanol application on insects

The insects exposed to *C. coronatus* SAB-GM cultures demonstrated the same effects as those described in previous works^9,33,36^. Among the *G. mellonella*, mortality was high among the larvae 48 h after treatment (over 90%) but very low among the adults (below 10%); for *C. vicina*, the larvae demonstrated complete resistance, but the adults displayed high mortality (over 95%) after three days: this has been attributed to their licking the surface of the fungal colonies. Also, the infected *G. mellonella* larvae demonstrated the same symptoms as described previously: dark spots on the cuticle, cessation of silk spinning and immobilization.

**Figure 2:**
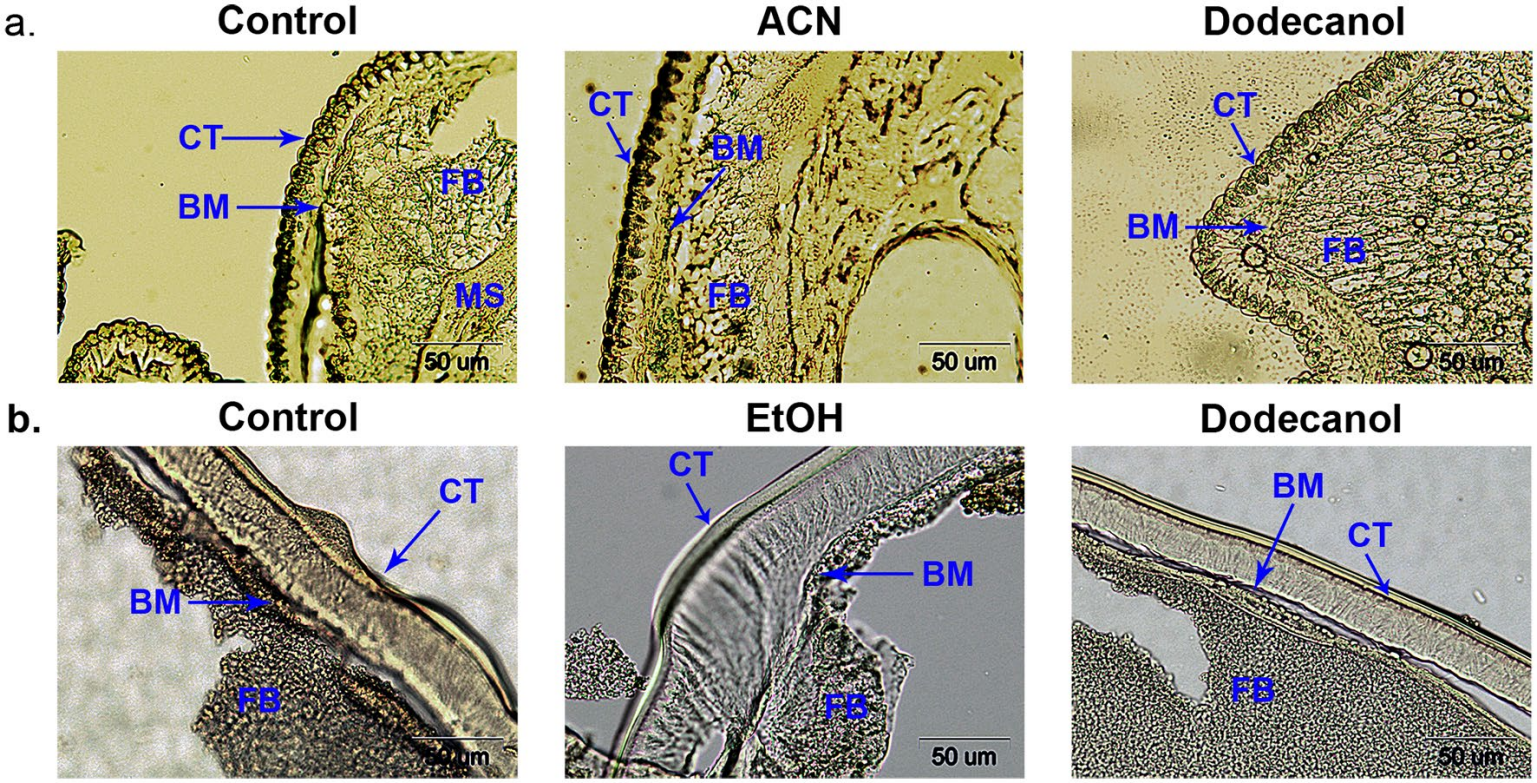
Light microscopy photographs of longitudinal sections through the last-instar larvae of (**a**) *Galleria mellonella* and (**b**) *Calliphora vicina* examined using the TUNEL method. *ACN* Acetone, *EtOH* Ethanol, *CT* Cuticle, *BM* Basement membrane, *FB* Fat body, *MS* Muscles.

Our present findings confirm that *C. vicina* larvae are not only resistant to fungal invasion but also to dodecanol treatment (Table 1). Mortality after dodecanol application was 10% in both larvae and adult flies. Despite the low mortality, in the larvae, dodecanol treatment impaired their further development, manifested by a prolonged pupal stage from 10.19 ± 0.99 days to 13.00 ± 1.44 days (Student’s t-test, df = 2, *p* = 0.008). It should be noted that only 30% of treated individuals reached the imago stage, while 89% of adults emerged in the untreated controls and 90% in the ethanol-treated controls (χ^2^ = 197,4, df = 4, *p* < 0,001). Among the *G. mellonella*, dodecanol treatment caused much higher mortality among adult moths (57%) than larvae (3.3%) (Table 1). Topical application of dodecanol did not exert any effect on the behavior or morphology of the treated insects. Histological examination of the dodecanol-treated insects did not reveal any changes in the morphology of the cuticle, basement membrane, muscles, fat body, Malpighian tubules or other studied vital organs and tissues. TUNEL staining did not reveal any apoptotic changes in the main body structures of the dodecanol-treated *G. mellonella* or *C. vicina* (Fig. 2).

**Table 1:** Effect of dodecanol on mortality of *Galleria mellonella* and *Calliphora vicina* larvae and adults.

Treatments	Percent (%) of dead insects (48 haa)			
	*G. mellonella* larvae	*G. mellonella* adults	*C. vicina* larvae	*C. vicina* adults
Untreated control	0	0	0	0
ACN/EtOH control	3.3 ± 4.71	10 ± 8.16	0	0
Dodecanol	3.3 ± 4.71	57 ± 26.25	10 ± 0.1	10 ± 0.1

All experiments were performed independently in triplicate, with approximately 15 individuals per replicate; results were showed as % of mortality ± SD (standard deviation).

*ACN* Acetone, *EtOH* Ethanol, *haa* Hours after application.

### The effect of dodecanol on larval hemocytes and Sf9 cultured in vitro

In vitro microscopic examination of *G. mellonella* hemolymph found that the treatment appeared to influence five immune cell types: prohemocytes, plasmatocytes, granulocytes, spherulocytes and oenocytoids. After 24 h of cultivation, the prohemocytes disappeared, the spherulocytes were present as floating cells, and the plasmatocytes formed a characteristic network with the granulocytes. In vitro microscope inspection of *C. vicina* larval hemolymph showed the presence of four types of hemocytes (according to Zachary and Hoffman)^37^: oenocytoids, plasmatocytes, type III plasmatocytes (or granulocytes) and thrombocytoids. *C. vicina* immune cells also formed a network after 24-h cultivation.

**Figure 3:**
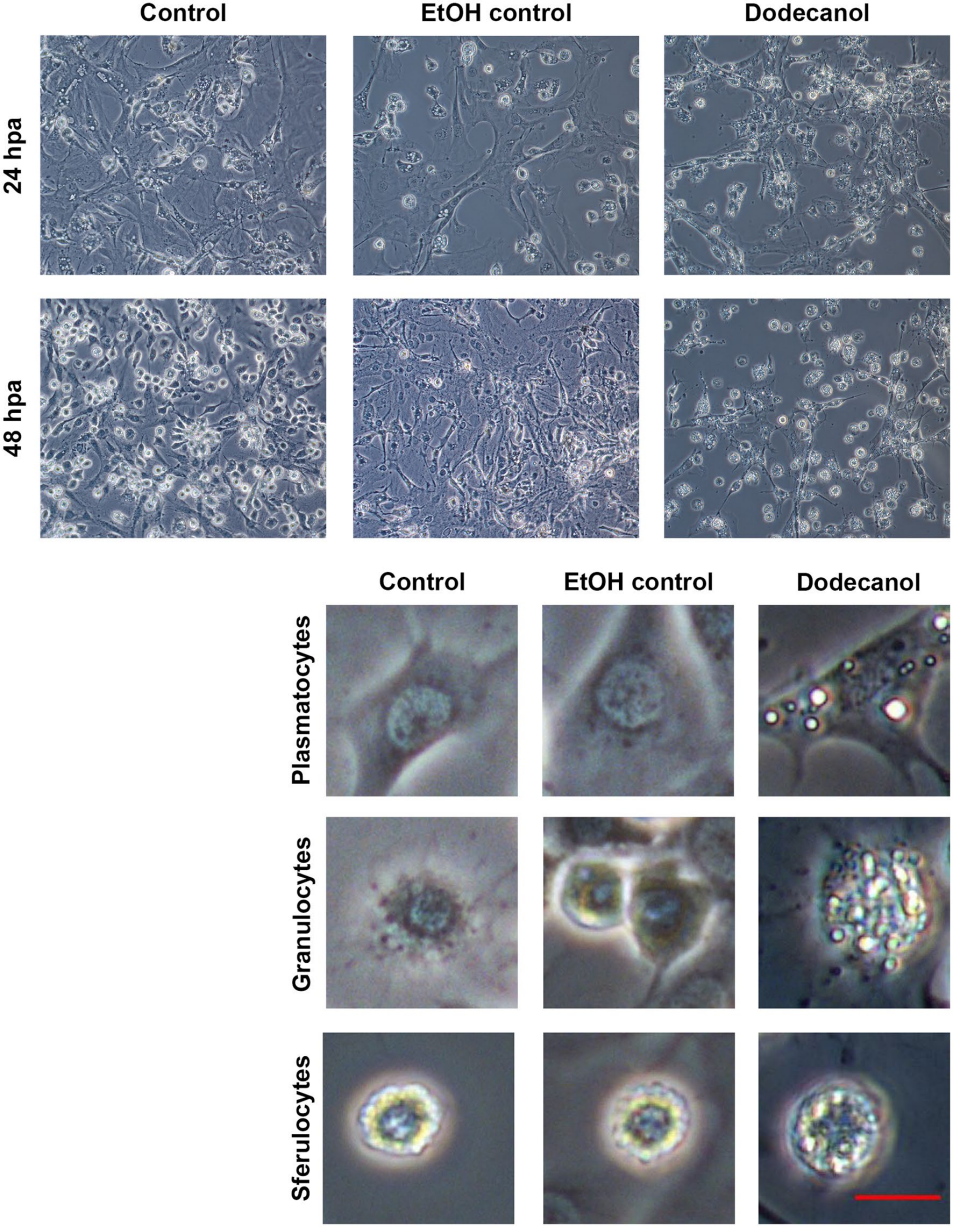
In vitro culture of hemocytes obtained from *Galleria mellonella* wax moth larvae. Control, ethanol control (1 µl 99.8% ethanol, final concentration 2.25 µg/µl) and dodecanol (final concentration 0.28 µg/µl) treated hemocytes. 24hpa—group 24 h after dodecanol application, 48hpa—group 48 h after dodecanol application. Scale bar 40 µm. Individual cell classes present in *G. mellonella* larvae hemocoel: control, ethanol control and 48 h after dodecanol application. Scale bar 10 µm.

**Figure 4:**
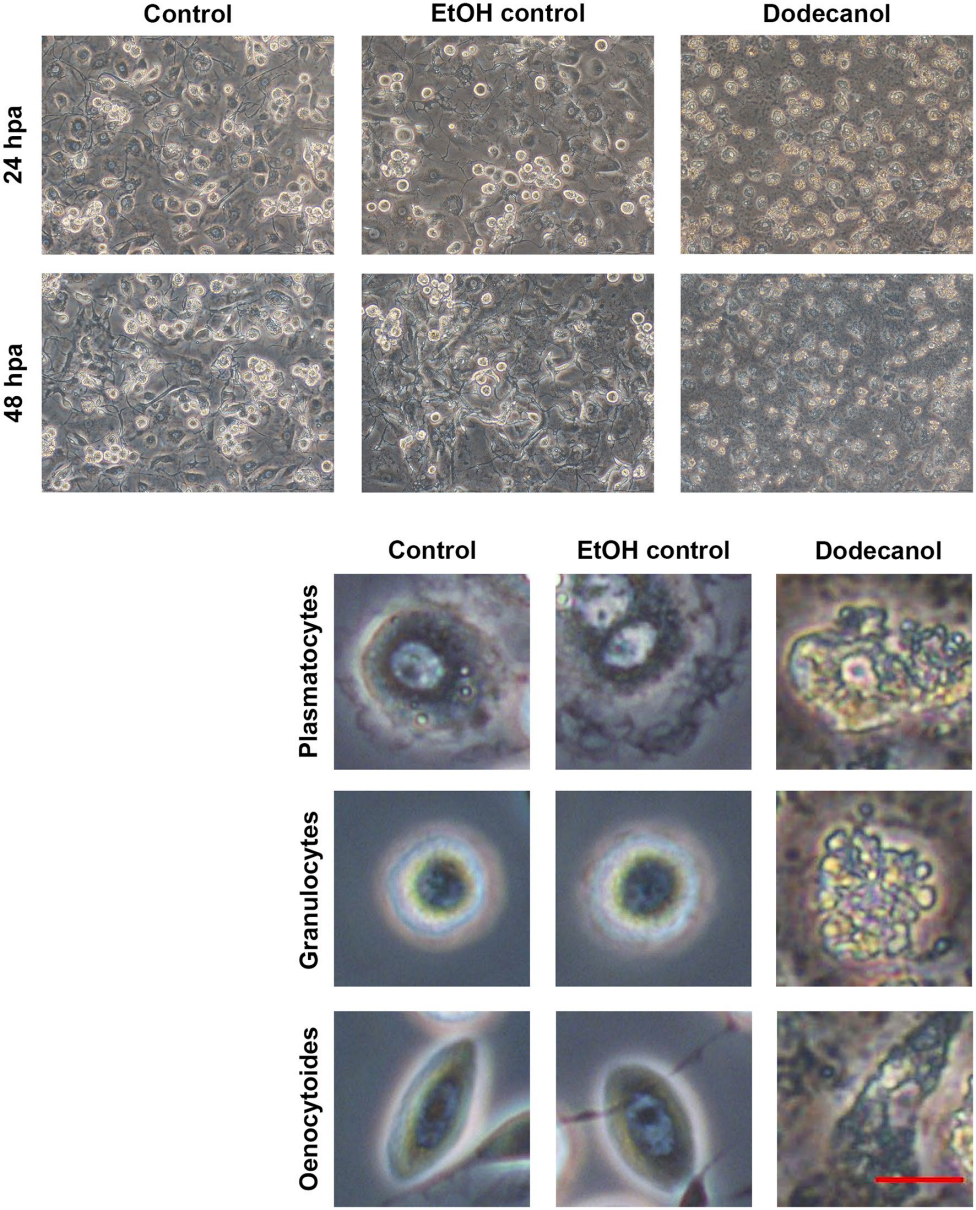
In vitro culture of hemocytes obtained from *Calliphora vicina* blow fly larvae. Control group, ethanol control group (1 µl 99.8% ethanol, final concentration 2.25 µg/µl) and dodecanol group (final concentration 0.28 µg/µl)-treated hemocytes. 24hpa—group 24 h post dodecanol application, 48hpa—group 48 h post dodecanol application. Scale bar 20 µm. Individual cell classes present in *C. vicina* larvae hemocoel: control group, ethanol control group and the group 48 h after dodecanol application. Scale bar 10 µm.

**Figure 5:**
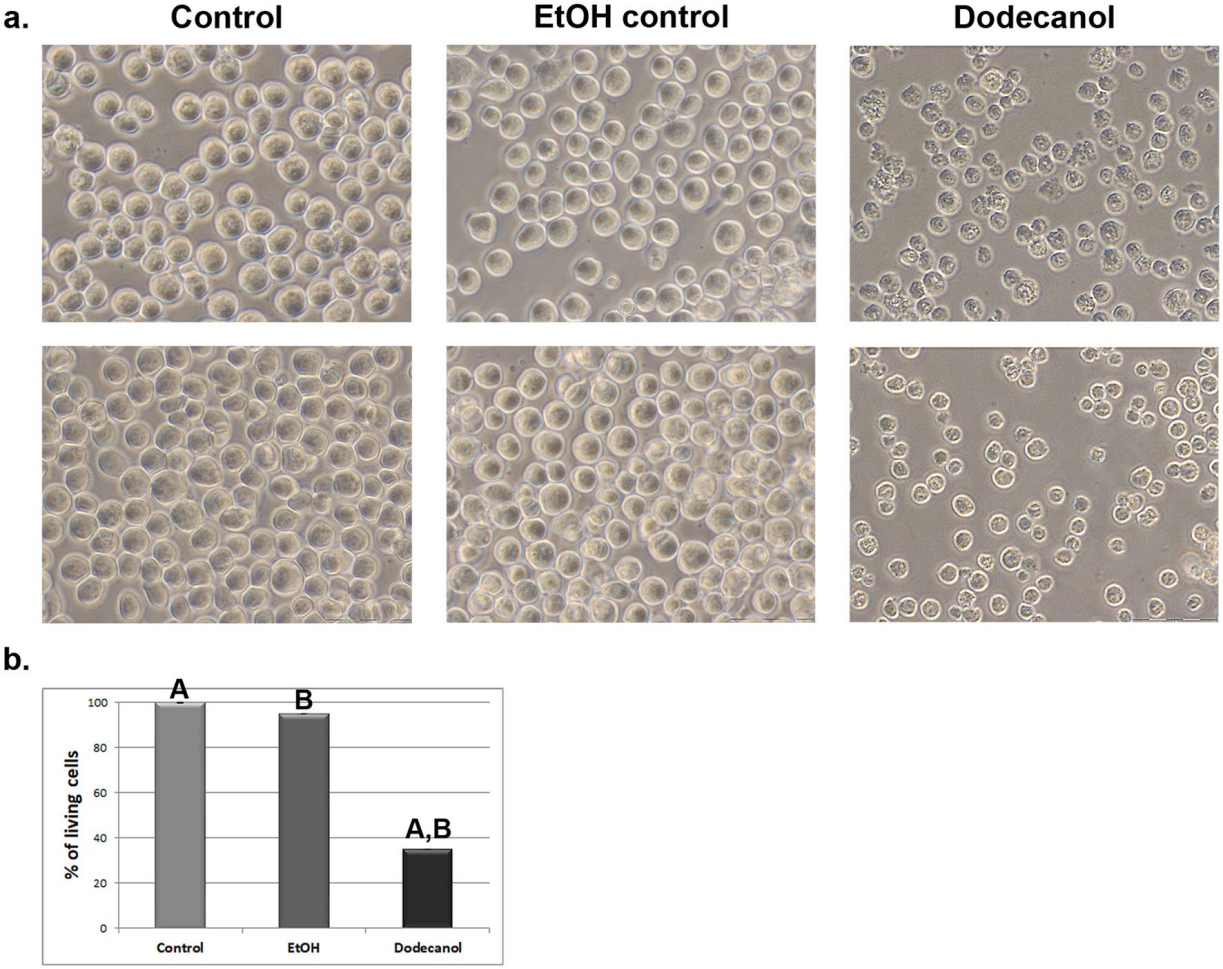
The morphological (**a**) and livability (**b**) changes in Sf9 cells after dodecanol treatment (**a**) Sf9 control group, ethanol control group (1 µl 99.8% ethanol, final concentration 2.25 µg/µl) and dodecanol group (final concentration 0.28 µg/µl) treated cell line. 24hpa—group 24 h after dodecanol application, 48hpa—group 48 h after dodecanol application. Scale bar 20 µm. (**b**) graph showing percent of living Sf9 cells 24 h after administration of dodecanol (final concentration 0.28 µg/µl), obtained using the WST-1 cell proliferation assay.

The effect of dodecanol on the hemocytes and Sf9 cell morphology of both insect species was documented at 24 and/or 48 h after application. Dodecanol demonstrated an inhibitory effect with high toxicity to all tested types of cells (Figs. 3, 4, 5). It was found that 24 h after metabolite administration, the hemocyte cultures demonstrated strong cell disintegration, inhibition of network formation by plasmatocytes and granulocytes, and lack of adhesion (plasmocytes, thrombocytoids), together with a very large number of dead cells. Furthermore, almost complete disintegration of all three types of tested cells was observed after 48 h.

Control cultures of *G. mellonella* larval hemocytes as well as dodecanol-treated hemocytes are shown in Fig. 3. Dodecanol treatment resulted in changes in cell shape and disturbances in the production of networks between cells; it also resulted in poor adhesion of hemocytes to the plate surface. A loss of intercellular networks was observed, together with cell disintegration; in addition, numerous vacuoles were visible in the cells. *C. vicina* larval hemocytes turned out to be more sensitive (Fig. 4), with severe cell damage, marked degranulation and lack of adhesion being observed after just 24 h, and most of the cells were dead and disintegrated after 48 h.

The Sf9 cells treated with dodecanol also demonstrated significantly greater shape changes and cell degranulation compared to controls (Fig. 5), with many being destroyed and disintegrated. The WST-1 test confirmed that dodecanol is toxic to Sf9 cells, with only 35% of the cells still alive 24 h after dodecanol application. As insect immune cells appear unable to proliferate in vitro, the WST-1 test was not performed on the *G. mellonella* or *C. vicina* hemocytes.

### The influence of dodecanol on cuticular FFA

**Figure 6:**
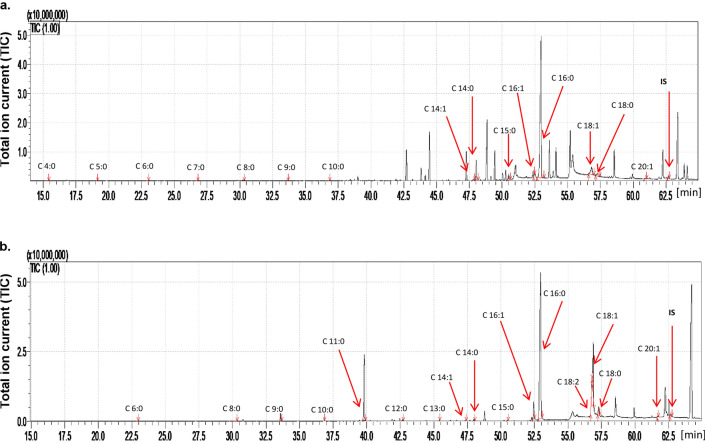
The total ion current (TIC) of fatty acids (TMS esters) of the dichloromethane extract from control *Galleria mellonella* (**a**) larvae and (**b**) imago. IS—internal standard—19-methylarachidic acid; fatty acids and molecular ions: butanoic acid (C4:0, m/z = 145), pentanoic acid (C5:0, m/z = 159), hexanoic acid (C6:0, m/z = 173), heptanoic acid (C7:0, m/z = 187), octanoic acid (C8:0, m/z = 201), nonanoic acid (C9:0, m/z = 215), decanoic acid (C10:0, m/z = 229), undecanoic acid (C11:0, m/z = 243), dodecanoic acid (C12:0, m/z = 257), tridecanoic acid (C13:0, m/z = 271), tetradecenoic acid (C14:1, m/z = 283), tetradecanoic acid (C14:0, m/z = 285), pentadecanoic acid (C15:0, m/z = 299), hexadecenoic acid (C16:1, m/z = 311), hexadecanoic acid (C16:0, m/z = 313), octadecadienic acid (C18:2, m/z = 337), octadecenoic acid (C18:1, m/z = 339), octadecanoic acid (C18:0, m/z = 341), eicozenoic acid (C20:1, m/z = 367).

GC/MS analysis indicated both quantitative and qualitative differences in cuticle FFA content between control *G. mellonella* and those treated with dodecanol (Table 2, Supplementary Table 2, Fig. 6). The total cuticular FFA content in control larvae was 485.49 µg/g of body mass, and 15 compounds from C4:0 to C20:1 were identified; of these, the highest concentrations were observed for C14:0, C16:0 and C18:1 (Table 2, Fig. 6). The control adults demonstrated significantly higher total cuticular FFA content, i.e. 6417.28 µg/g of body mass (13.2 times higher than treated adults; *p* = 0.000291, df = 4) and 16 FFAs were found from C6:0 to 20:1 (Table 2). Among the controls, the untreated larvae demonstrated three short-chain cuticular FFAs that were absent from the imagoes: C4:0, C5:0 and C7:0. Conversely, four FFAs absent from control larvae were found to be present in in control adults: C11:0, C12:0, C13:0, and C18:2. It should be noticed that the FFAs identified in both developmental stages were present at higher concentrations in adults than in larvae (Table 2).

**Table 2:** Content of free fatty acids present on the cuticle of *Galleria mellonella* larvae and adults (µg/g of body mass ± SD).

FFA	*Galleria mellonella* larvae (µg/g of body mass ± SD)			*Galleria mellonella* adults (µg/g of body mass ± SD)		
	Control	ACN	Dodecanol	Control	ACN	Dodecanol
C_4:0_	0.72 ± 0.28	ND	ND	ND	ND	ND
C_5:0_	0.36 ± 0.13	0.29 ± 0.13	ND	ND	ND	ND
C_6:0_	1.11 ± 0.47	1.30 ± 0.37	2.25 ± 0.51	2.04 ± 1.03^a^	0.63 ± 0.28^a^	0.25 ± 0.14^a^
C_7:0_	0.96 ± 0.34	1.17 ± 0.71	ND	ND	0.38 ± 0.33	ND
C_8:0_	1.63 ± 0.05	2.28 ± 1.01	3.65 ± 1.14	4.36 ± 1.47^b^	1.31 ± 0.6^b^	ND
C_9:0_	1.34 ± 0.17	2.44 ± 0.74^A^	4.01 ± 1.04^A^	112.02 ± 6.48^c^	56.97 ± 16.53^c^	0.98 ± 0.02^c^
C_10:0_	0.96 ± 0.30	0.68 ± 0.3	ND	4.37 ± 3.18	0.64 ± 0.32	ND
C_11:0_	ND	ND	ND	1268.38 ± 361.77^d^	432.38 ± 126.11^d^	32.84 ± 3.33^d^
C_12:0_	ND	ND	ND	23.94 ± 5.77^e^	2.40 ± 1.14^e^	29.96 ± 1.82
C_13:0_	ND	ND	ND	7.92 ± 0.25f.	3.58 ± 1.51	0.91 ± 0.17f.
C_14:1_	2.02 ± 1.35	ND	ND	12.12 ± 6.61	ND	ND
C_14:0_	28.69 ± 9.39^B^	16.59 ± 2.45^C^	63.01 ± 4.6^B,C^	43.94 ± 13.15^j^	6.83 ± 2.37^j^	3.31 ± 0.33^j^
C_15:0_	7.12 ± 5.79	9.26 ± 4.41^E^	19.99 ± 4.58^E^	17.93 ± 11.24^ l^	3.25 ± 1.57^ l^	3.82 ± 5.41^ l^
C_16:1_	18.34 ± 13.72	1.05 ± 0.33^F^	87.06 ± 11.92^F^	264.61 ± 9.36	29.98 ± 12.87	150.31 ± 242.56
C_16:0_	329.52 ± 154.13^G^	842.77 ± 38.43^H^	3137.41 ± 188.82^G,H^	3687.79 ± 323.09^o^	1057.30 ± 120.18^o^	285.02 ± 249.94^o^
C_18:2_	ND	ND	ND	62.52 ± 84.21^p^	14.28 ± 5.46^p^	99.83 ± 6.64^p^
C_18:1_	80.19 ± 49.57^I^	57.25 ± 17.89^ J^	343.37 ± 23.51^I,J^	730.16 ± 142.87^ s^	344.56 ± 30.01^ s^	414.23 ± 33.46^ s^
C_18:0_	12.27 ± 2.72	ND	ND	168.87 ± 10.78	65.36 ± 27.82	71.26 ± 2.38
C_20:1_	1.33 ± 0.37	ND	ND	6.29 ± 2.83	7.38 ± 2.15^u^	30.59 ± 2.69^u^
Sum of FFAs	485.49 ± 238.78	934.79 ± 66.78	3662.35 ± 236.68	6417.28 ± 984.12	2027.21 ± 349.28	1123.31 ± 548.93

Statistically significant differences are marked with the same letters (capital letters were used for larvae and small letters for adults) (ANOVA, Tukey’s HSD Test, *p* < 0.05).

*SD* Standard deviation, *ACN* Acetone control, *FFA* Free fatty acid, *ND* Not detected.

Treatment of *G. mellonella* with dodecanol resulted in a 7.5 fold increase in total cuticular FFA content in larvae (*p* = 0.000298, df = 4), and a 5.7 fold decrease in adults (*p* = 0.000291, df = 4). Solvent application, i.e. acetone only, resulted in a 1.9 fold elevation of total FFA content in larvae (*p* = 0.00274, df = 4) and 3.2 fold drop in adults (*p* = 0.000296, df = 4). In the larvae, dodecanol application resulted in the loss of C4:0, C5:0, C7:0, C10:0, C14:1, C18:0 and C20:1; however, it should be noted that C4:0, C14:1, C18:0 and C20:1 also disappeared after the application of solvent (acetone) alone, so only the loss of C5:0, C7:0, C10:0 can be treated as a true effect of dodecanol. In addition, dodecanol treatment resulted in an increase in C14:0, C15:0, C16:1, C16:0 and C18:1 in larvae (*p* < 0.001, df = 4); however, C16:0 only increased after acetone treatment (Table 2).

Similarly, dodecanol treatment resulted in quantitative and qualitative changes in the cuticular FFA profile of the adult insects. Treatment resulted in the disappearance of C8:0, C10:0 and C14:1, the latter probably due to the solvent, as well as a significant decrease of C6:0, C9:0, C11:0, C13:0, C14:0, C15:0, C16:0, and C18:1 compared with controls (*p* < 0.001, df = 4); in all these cases, the reduction was partly due to acetone. The only FFA to increase was C20:1. An interesting response to the acetone administration was the appearance of a small amount of C7:0, which was absent in untreated moths. Surprisingly, while the levels of C12:0, C16:1 and C18:0 fell in acetone-treated adults, no such change was observed in those treated with the dodecanol mixture, even though both groups of insects received the same amount of acetone (Table 2).

**Figure 7:**
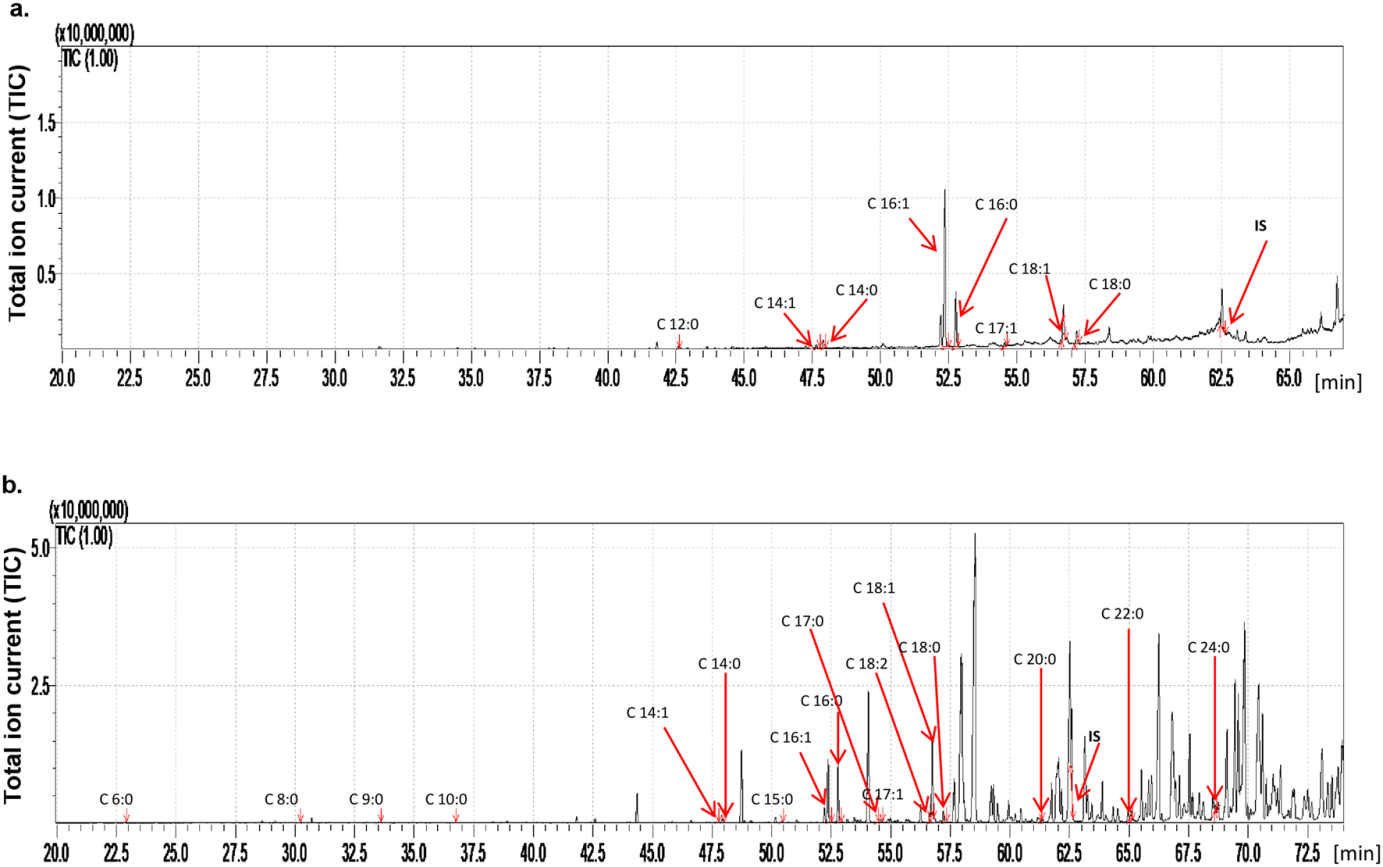
The total ion current (TIC) of fatty acids (TMS esters) of the dichloromethane extract from control (**a**) *Calliphora vicina* larvae and (**b**) imago. IS—internal standard—19-methylarachidic acid; fatty acids and molecular ions: hexanoic acid (C6:0, m/z = 173), octanoic acid (C8:0, m/z = 201), nonanoic acid (C9:0, m/z = 215), decanoic acid (C10:0, m/z = 229), dodecanoic acid (C12:0, m/z = 257), tetradecenoic acid (C14:1, m/z = 283), tetradecanoic acid (C14:0, m/z = 285), pentadecanoic acid (C15:0, m/z = 299), hexadecenoic acid (C16:1, m/z = 311), hexadecanoic acid (C16:0, m/z = 313), octadecadienic acid (C18:2, m/z = 337), heptadecenoic acid (C17:1, m/z = 325), heptadecanoic acid (C17:0, m/z = 328), octadecadienic acid (C18:2, m/z = 337), octadecenoic acid (C18:1, m/z = 339), octadecanoic acid (C18:0, m/z = 341), eicozenoic acid (C20:1, m/z = 367), docosanoic acid (C22:0, m/z = 397), tetracosanoic acid (C24:0, m/z = 425).

Compared with *G. mellonella*, the *C. vicina* larvae yielded 10.8 times less total FFA content (*p* = 0.002146, df = 4) and the adults 72.8 times lower (*p* = 0.000291, df = 4) (Tables 2, 3, Figs. 6, 7). As with *G. mellonella*, more FFAs were found in adults than in larvae, but the difference was much lower, only 1.97 times (*p* < 0.0001, df = 4). Only eight saturated and unsaturated FFAs from C12:0 to C18:0 were identified in the larvae, while 17 FFAs from C6:0 to C26:0 were found in adult flies. In the control adults, 10 FFAs were observed that were absent from control larvae: C6:0, C8:0, C9:0, C10:0, C15:0, C17:0, C18:2, C20:0, C22:0 and C24:0. Conversely, C12:0 was found in larvae but not in adults (Table 3, Fig. 7). The acids common to untreated larvae and adults were found in similar concentrations (C14:1, C14:0, C16:1, C17:1, C18:0); however, the level of C16:0 was 2.2 times higher (*p* < 0.001, df = 4) in adults and C18:1 was 4.5 times higher (*p* < 0.001, df = 4) (Table 3).

**Table 3:** Content of free fatty acids present on the cuticle of *Callihopra vicina* larvae and adults (µg/g of body mass ± SD).

FFA	*Calliphora vicina* larvae (µg/g of body mass ± SD)			*Calliphora vicina* adults (µg/g of body mass ± SD)		
	Control	EtOH	Dodecanol	Control	EtOH	Dodecanol
C_6:0_	ND	ND	ND	0.09 ± 0.02	0.09 ± 0.01	ND
C_8:0_	ND	ND	ND	0.12 ± 0.01	0.21 ± 0.04	ND
C_9:0_	ND	ND	ND	0.45 ± 0.02^d^	0.37 ± 0.01^d^	0.26 ± 0.04 ^d^
C_10:0_	ND	ND	ND	0.13 ± 0.02f.	0.15 ± 0.01	0.36 ± 0.10f.
C_11:1_	ND	ND	ND	ND	1.33 ± 0.08	0.73 ± 0.21
C_11:0_	ND	ND	2.88 ± 0.59	ND	ND	ND
C_12:0_	0.25 ± 0.13^A^	0.91 ± 0.06^B^	56.14 ± 2.64^A,B^	ND	ND	ND
C_13:0_	ND	ND	ND	ND	ND	ND
C_14:1_	0.49 ± 0.23	0.61 ± 0.03	ND	0.39 ± 0.06^ h^	1.93 ± 0.69^ h^	2.06 ± 0.26^ h^
C_14:0_	1.06 ± 0.48	2.01 ± 0.10	ND	1.41 ± 0.06^j^	7.59 ± 0.45	13.69 ± 4.99^j^
C_15:0_	ND	0.63 ± 0.02	ND	0.49 ± 0.01	1.24 ± 0.22	1.46 ± 0.58
C_16:1_	24.46 ± 3.19^F^	39.88 ± 2.27^G^	89.85 ± 7.95^FG^	23.84 ± 1.57^n^	138.64 ± 7.56^n^	178.88 ± 62.41^n^
C_16:0_	9.73 ± 0.82^H^	21.43 ± 1.41	47.47 ± 4.28^H^	21.81 ± 0.85^p^	86.38 ± 5.39^p^	113.66 ± 39.50^p^
C_17:1_	0.55 ± 0.08^ J^	1.76 ± 0.06^ K^	3.16 ± 0.78^ J,K^	0.19 ± 0.02^ s^	1.24 ± 0.06^ s^	1.44 ± 0.56^ s^
C_17:0_	ND	0.36 ± 0.18	ND	0.77 ± 0.12^t^	5.49 ± 0.34^t^	5.99 ± 2.16^t^
C_18:2_	ND	ND	ND	2.19 ± 0.05^u^	19.93 ± 1.61^u^	9.43 ± 3.16^u^
C_18:1_	6.04 ± 0.49^L^	18.59 ± 1.07	37.06 ± 5.06^L^	26.98 ± 1.61^w^	144.21 ± 6.22^w^	173.81 ± 62.81^w^
C_18:0_	2.23 ± 0.27	3.57 ± 0.16	7.31 ± 1.37	3.82 ± 0.05	11.75 ± 0.85	12.86 ± 4.93
C_20:0_	ND	ND	ND	0.85 ± 0.55	0.36 ± 0.03	ND
C_22:0_	ND	ND	ND	2.88 ± 0.04	2.16 ± 0.13	ND
C_23:1_	ND	0.90 ± 0.19	ND	ND	ND	ND
C_23:0_	ND	1.54 ± 0.21	ND	ND	ND	ND
C_24:0_	ND	ND	ND	1.71 ± 0.19	1.90 ± 0.17	ND
C_26:0_	ND	ND	ND	ND	4.45 ± 0.44	ND
Sum of FFAs	44.82 ± 5.70	92.20 ± 5.76	243.87 ± 23.23	88.13 ± 5.29	429.47 ± 24.32	514.65 ± 181.74

Statistically significant differences are marked with the same letters (capital letters were used for larvae and small letters for adults) (ANOVA, Tukey’s HSD Test, *p* < 0.05).

*SD* Standard deviation, *EtOH* Ethanol control, *FFA* Free fatty acid, *ND* Not detected.

Dodecanol administration resulted in 5.4 times (243.87 µg/g of body mass) higher content of total FFAs in larvae (*p* = 0.000357, df = 4) and 5.8 times (514.65 µg/g of body mass) higher in adults (*p* = 0.0152, df = 4) compared with their respective controls. However, in both cases, the application of solvent (ethanol) also induced an elevation of total FFAs: 2.1 times in larvae (*p* = 0.000374, df = 4) and 4.9 times in adults (*p* = 0.000293, df = 4). Following dodecanol treatment the larvae lost C14:1 and C14:0 from the cuticle, but gained C11:0 and an increase in the levels of C12:0 and C17:1. This increase in FFA level may have been partly due to the solvent, as ethanol application also increased content of these FFAs, albeit to a lesser extent. In adult flies, the same treatment resulted in the disappearance of C6:0, C8:0, C20:0, C22:0 and C24:0 and the appearance of C11; however, in this case, its appearance was probably caused by the ethanol rather than by dodecanol (Table 3). Ethanol treatment also contributed to the dodecanol-induced elevation of C14:1, C16:1, C16:0, C17:1, C17:0, and C18:2 (Table 3).

## Discussion

The growing resistance of insect pests forces the search for new insecticides that are safe for humans and the environment. Previous studies at the Institute of Parasitology, the Polish Academy of Sciences, have identified several insecticidal proteins produced by *C. coronatus*, two of which are coronatin-1 (36 kDa) and coronatin-2 (14.5 kDa). Both coronatins kill *G. mellonella* larvae and influence the functioning of hemocytes, although in different ways^19,23,25^. In addition, a serine protease with a mass of 30–32 kDa, with subtilisin properties, was also detected^38^. Preliminary studies also suggest that this fungus is capable of producing other proteins which may also have insecticidal properties. The toxicity of another four proteins secreted by *C. coronatus* to the incubation medium is discussed by Samborski (PhD thesis)^39^. Our team has also recently identified several dozen substances produced by *C. coronatus* which are currently being tested for insecticidal potential^26,27,39,40^. One such substance is dodecanol, which has already been found to demonstrate high insecticidal activity against various species including *Lucilia serica* and *Musca demestica* (^40^; unpublished data).

The current experiments used two different species of insect from two different orders. The greater wax moth *G. mellonella* is commonly used as a model host for studying human pathogens^4,33,41,42^ as it is cheap and easy to maintain, and tolerates a breeding temperature of 37 °C^43^. The second model insect used in the experiments was *C. vicina*, whose larvae are extremely resistant to fungal infections and hence are interesting subjects for research into the mechanisms of antifungal immunity^9^. Adult flies can act as vectors for various pathogenic microorganisms and are important in forensics.

Previous research into the infection of *C. coronatus* of *G. mellonella* showed that after fungal infection, wax moth larvae became immobilized, are unable to construct cocoons, and cease silk spinning, which is continuously produced in normal conditions^36^. Furthermore, after exposure to the fungal colony, black spots develop across the surface of the cuticle, indicating sites of fungal penetration. These spots spread until the cuticle of the dying insects becomes completely black, and approximately 48 h later, the larvae die^33,36^. The changes described above also occurred in the present study. *C. vicina* was more resistant to fungal infection after exposure to sporulating fungal colonies; however, previous studies note that injection of *C. coronatus* conidia resulted in 100% mortality in 24 h^9^. The *C. vicina* exposed to the fungus did not demonstrate any signs of fungal penetration through the fly cuticle, nor any changes to the internal organs, nor any mobilization of haemocytes to eliminate the fungal pathogen; in contrast, those injected with *C. coronatus* conidia suffered profound damage to the internal organs, which suggest that structure of the host exoskeleton, particularly the composition of lipids present on the cuticle, seems to be major factor determining the susceptibility or resistance of insect species to *C. coronatus* infection^1,3,4,6,6–8,34,44^. The possibility that the cuticle plays a pivotal role in ensuring resistance to fungal infection in Dipteran flies is supported by further observations that *C. vicina* larvae have thicker cuticles than those of *G. mellonella* or *D. pini*, which are also more susceptible to fungal infection^9^. The role of cuticular FFAs in resistance to fungal infection by *C. vicina*, *D. pini* and *G. mellonella* larvae has also been described by Gołębiowski et al.^8^.

The cuticle could play also an important role in intoxication by dodecanol, either as an external target or as an initial barrier to an internal target. Topical application of a high dose of 1-dodecanol (1 µg/insect) on teneral first nymphs of *Rhodnius prolixus* caused an interruption of the darkening process of the cuticle, leading to the dehydration and the death of the treated nymph^45^. Moreover, it was found that the lipophilicity and dehydrating properties of the dodecanol determined its capability to penetrate the cuticle of the eggs, larvae and pupae of *Aedes aegyti* as well as, leading to the death of the embryo or the larvae; it is believed that it acts by breaking down the lipid composition of the target^46^. Dodecanol also demonstrated high insecticidal activity against *Pediculus humanus capitis* and enhanced the pediculicidal activity of d-phenothrin by enhancing the penetration of the insecticide through the cuticle^47^.

Even so, dodecanol is generally recognized as safe (GRAS). It is permitted as a food additive in both the U.S. and the EU, and is already registered for use as a pesticide in the U.S. as a pheromone/sex attractant, used to disrupt the mating behavior of Lepidopteran species whose larvae destroy crops^48^. Existing studies report the presence of dodecanol in female *Cydia pomonella* insects, suggesting it may have a role as a pheromone^49,50^.

Several organisms including *Escherichia coli* produce dodecanol^51^; however, as synthesis from olefins requires high temperature and pressure and the use of heavy metal catalysts, industrial production is derived mainly from palm kernel oil and coconut oil. However, biocatalytic methods using recombinant *E. coli* strains with an introduced alkane-inducible monooxygenase operon that can efficiently catalyze the conversion of alkane to 1-alkanol have recently gained attention because of their high stereo- and regioselectivities and mild reaction conditions^52^. The pathway of dodecanol synthesis by the fungus *C. coronatus* remains unknown, but it will hopefully soon be revealed by the full sequencing of its genome^53^. It should be emphasized that it remains unclear whether this compound is produced by the hyphae spreading inside the infected insects. This is a subject planned for future studies.

It is also unclear whether the dodecanal, after being applied to the surface of the insect body and passing through the cuticle, is passively or actively transported inside the host, and its methods of distribution and metabolism remain unknown. Frog liver microsomes catalyze the hydroxylation of 1-dodecanol into the corresponding omega- and (omega-1)-hydroxy derivatives involving cytochrome P-450; a fatty alcohol oxidation system may also play a role, as laurate formation has been observed in frog liver microsomes. 1-Dodecanol was also hydroxylated very effectively by gerbil liver microsomes, but in general, the hydroxylation rates for fatty alcohols were much lower than those for the corresponding acids^54^. More research is needed to see if dodecanol is metabolized in a similar way in the fat body of insects, which is the functional counterpart of the vertebrate liver.

The penetration through the cuticle of the tested substances is an important issue; it often causes technical problems and can have an influence on the obtained results. The efficiency of dodecanol penetration through the *G. mellonella* and *C. vicina* cuticle remains unknown; it is also unclear whether such penetration differs between species and between larvae and adults due to the fundamentally different structures of their cuticles. The lack of morphological changes in the internal organs and tissues of insects treated with dodecanol, as well as the lack of any noticeable changes in behavior, might suggest either a rapid metabolism or a good tolerance of this compound. On the other hand, the failure of most *C. vicina* larvae to reach the imago stage following dodecanol treatment may indicate that it has a long-term effect on the mechanisms involved in directing the correct course of metamorphosis.

The choice of solvent plays a key consideration in the topical application procedure. The present study used acetone for *G. mellonella* and ethanol for *C. vicina*. Acetone is believed to possess relatively low acute toxicity for mammals, and for aquatic and terrestrial arthropods, and is frequently used in physiological and biochemical investigations on insects^55^. Acetone metabolized by carboxylation to acetoacetate, as shown in bacteria, is incorporated into fatty acids during lipogenesis in mammal adipose tissue^56–58^.

Presently, no data exists on the metabolism of acetone in insects or the presence of any enzymatic machinery that can convert acetone to FFAs in insect tissues. More is known about the metabolism of ethanol and its impact on lipid metabolism in insects. The dependence of the flux in the alcohol-degrading pathway on the activity of alcohol dehydrogenase (ADH), the initial enzyme in the key ethanol-degrading pathway, was investigated in *Drosophila melanogaster* with different ADH variants^59^. Dietary ethanol reduced the chain length of total FFAs and reduced the saturation of short-chain FAAs in larvae with functional ADH; however, in ADH-null mutants, ethanol promoted an increase in the length of total FAAs. This finding suggests that while the effect of ethanol on the change in FAA length was ADH dependent, its effect on FAA desaturation was not. Ethanol also stimulates a decrease in the relative amount of phosphatidylcholine and an increase in phosphatidylethanolamine in *D. melanogaster* membrane lipids, similarly to that described in other animals^60^.

Mayoral and co-workers indicate that *Aedes aegyptii* short-chain dehydrogenase 9 (AaSDR9), an enzyme which plays critical roles in lipid and xenobiotic metabolism, has high preference for dodecanol and displays a 70-fold increase in activity when it is used as a substrate^61^.

The above data should be taken into account when assessing the effects of dodecanol administration on the cuticle lipid profiles. Our present findings indicate that topical application of dodecanol had profound effects on the cuticular FFAs profiles of the tested species, resulting in qualitative and quantitative changes, which were both species- and developmental stage-dependent.

In the wax moths, 15 different saturated and unsaturated fatty acids were identified in untreated larvae, and 16 in adults; however, in *C. vicina*, only eight FFAs were observed in larvae and 17 in adults. A number of saturated and unsaturated FFAs ranging from C6:0 to C20:1 were found in the cuticle of control *G. mellonella* larvae, and from C4:0 to C20:1 in the adults. FFAs C16:0 and C18:1 predominated in all developmental stages. FFAs from C11:0 to C26:0 were observed on the cuticle of *C. vicina* larvae, while C6:0 to C26:0 and C16:1, C16:0 and C18:1 predominated in the adults. Furthermore, the FFAs profiles of control *G. mellonella* and, *C. vicina*, are similar to those described previously^33,34,44,62^. *G. mellonella* belongs to a different order than *C. vicina*, and the differing profiles suggest that the composition of the insect cuticle is species specific. However, differences were observed between the developmental stages of the same species.

In the *G. mellonella* larvae, dodecanol applied in acetone to resulted in a 7.5-fold increase of total cuticular FFAs while acetone alone was responsible for a 1.9-fold increase compared to untreated larvae. In adult moths, dodecanol applied in acetone resulted in a 5.7-fold decrease in total cuticular FFAs and dodecanol alone in a 3.2-fold decrease. The disappearance of C5:0, C7:0 and C10:0 from the larval cuticle appears to be due to dodecanol, while acetone application was responsible for the disappearance of C4:0, C14:1, C18:0, and C20:1 (Table 2). Dodecanol with acetone resulted in the elevation of C16:0 level, while dodecanol alone elevated C14:0, C15:0, C16:1 and C18:1.

In the case of adults, dodecanol treatment resulted in the disappearance of C8:0 and C10:0, and acetone alone in the disappearance of C14:1. Dodecanol stimulated an increase in C20:1 concentration, while dodecanol with acetone resulted in a decrease in the concentration of C6:0, C9:0 C11:0,C13:0, C14:0, C15:0, C16:0, C18:1, and C18:0. Additional research is required to determine what mechanisms underlie these changes and how dodecanol and acetone affect the synthesis and degradation of individual FFAs, and their transport to and from the cuticle.

Dodecanol applied in ethanol to *C. vicina* larvae resulted in 5.4-fold increase of total cuticular FFAs while ethanol alone was responsible for a 2.1-fold increase, compared to untreated larvae. In adult flies, dodecanol with ethanol resulted in 5.8-fold increase in total cuticular FFAs, and dodecanol alone in a 4.9-fold rise. In larvae, dodecanol treatment alone resulted in the disappearance of C14:1 and C14:0 from the larval cuticle and the appearance of C11:0 (absent in control larvae), while those treated with ethanol alone demonstrated C15:0, C17:0, C23:1 and C23:0 (Table 3). Combined dodecanol and ethanol treatment resulted in elevated levels of C12:0, C16:1, C17:1, and C18:1 in the larval cuticle. In adult flies, dodecanol treatment alone resulted in the disappearance of C6:0, C8:0, C 20:0, C22:0, and C24:0 as well as elevation of C14:1 concentration, while dodecanol with ethanol resulted in elevation of C14:0, C16:1, C16:0, C17:1, C17:0, C18:2, C18:1, and C18:0. Ethanol treatment alone resulted in the appearance of C11:1 and C26:0, which were both absent in control flies.

It cannot be excluded that the rise in FFA concentration observed following dodecanol treatment might be associated with the destruction of the fat body and the release of FFAs from disintegrating hemocytes and other insect cells; this is particularly likely considering that dodecanol destroys the hemocytes of both studied insect species. It is possible that entirely different mechanisms govern lipid metabolism at different stages of development, as indicated by the fact that dodecanol and its solvents (acetone, ethanol) administered alone differentially influence the appearance or disappearance of individual FFAs in larval and imaginal cuticles.

Clearly, further research is required to solve these complex issues; the existing data on the influence of mycotoxins on the lipid metabolism is fragmentary, and only a relatively small amount of such work exists on insects. Fumonisin B1 and deoxynivalenol are known to display synergistic or additive effects in lipid peroxidation in mice^63^. Altertoxin II, a potent genotoxic compound from *Alternaria alternata*, also induces lipid peroxidation possibly streaming down at the mitochondrial level of THP-1 macrophages^64^. Aflatoxins induce peroxidation of membrane lipids, which reduces membrane integrity and induces cell disintegration; trichothecenes and T-2 toxin interfere with the metabolism of membrane phospholipids and increase liver lipid peroxidation; in addition, fumonisin activity disrupts the pathway of de novo sphingolipid biosynthesis by inhibiting the enzyme ceramide synthase. These activities induce a wide spectrum of changes in lipid metabolism and associated lipid-dependent signaling pathways known to disturb numerous cell functions^65^.

The cellular response to entomopathogens is mediated by insect immunocompetent cells called hemocytes, which can phagocytose and kill invading small pathogens and encapsulate larger ones^36,66,67^. The effects of *C. coronatus* infection and the activities of its selected metabolites have been studied previously on *G. mellonella* hemocytes^19,25–27,36,66^; however, in contrast to *G*. *mellonella*, the composition of hemocytes of *C. vicina* remains poorly understood. Nevertheless, they are believed to employ three classes of hemocytes, viz*.* plasmatocytes, oenocytoids and trombocytoids, with the plasmatocytes being further differentiated into several types depending on developmental stage^37^.

In the present study, phase contrast microscopy revealed the presence of oenocytoids, plasmatocytes and type III plasmatocytes (or granulocytes) and thrombocytoids in the hemocytes of the control *C. vicina*. Previous studies conducted by Boguś and co-workers found *C. vicina* to have lower cellular and humoral immunity potential comparing to *G. mellonella*, including reduced phagocytic and encapsulation response, low phenoloxidase and lysozyme-like activity; however, this was compensated for by investment in a thick cuticle, which efficiently protects fly larvae from fungal assault.

The present work documents the effect of dodecanol on hemocyte morphology after 24 and 48 h in vivo cultivation. All species-specific hemocyte types were visible in the control *G. mellonella* and *C. vicina* larvae, and the cellular networks were properly formed in vitro. Dodecanol application altered cytoskeleton organization and was found to have a toxic effect against the tested Sf9 cell line, and the *G. mellonella* and *C. vicina* hemocytes. As soon as 24 h after dodecanol treatment, changes in cell structure were observed: the cells became more rounded, and lacked a characteristic network for hemocytes. After 48 h, all three types of cells were found to have disintegrated, and hemocyte classification was almost impossible. The WST-1 test confirmed that dodecanol effectively killed the Sf9 cells. Previously, similar net and cell disintegration were also seen in hemocyte cultures originating from *C. coronatus* infected *G. mellonella* larvae^37^ suggesting that dodecanol plays a role in pathogenesis development. However, our present findings rule out the participation of dodecanol in the stimulation of apoptosis; in addition, no studies have been performed on its effect on insects or insect cells.

## Conclusion

Our present findings indicate that a metabolite of *C. coronatus*, dodecanol, is secreted into the medium in which the fungus grows; it kills *G. mellonella* imagoes, prevents most *C. vicina* larvae from successfully completing metamorphosis and reaching the imago stage, has a negative impact on the hemocytes of *G. mellonella* and *C. vicina*, as well as the Sf9 cell line, and alters the cuticular FFA profiles of both species. This opens up another possible research path related to the functioning of *C. coronatus* and its mechanism of action. It is worth emphasizing that these studies are pioneering. Even so, our findings have a practical aspect and can open up broad horizons for the possibility of creating a new class of bioinsecticides using entomopathogenic fungi and their metabolites. It is worth emphasizing that dodecanol can be used as a selective insecticide and is regarded as safe for human beings by the Organisation for Economic Co-operation and Development (OECD) and European Food Safety Authority (EFSA), even for use as a food additive.

Substances produced by insecticidal fungi do not have to be limited only to the production of insecticides. Cyclosporine A or beauverolides, which were initially isolated as substances to cope with pest insects, have also found applications in medicine: Cyclosporin A is currently one of the more important drugs used in transplantation, due to its immunosuppressive effects^68,69^, and beauverolides are promising anti-atherosclerotic substances^70,71^. It cannot be excluded that some bioactive metabolites of *C. coronatus* may also be used in medicine someday.

## Supplementary Information

Supplementary Table 1.

Supplementary Table 2.
