# Supplementary material for: Dodecanol, metabolite of entomopathogenic fungus Conidiobolus coronatus, affects fatty acid composition and cellular immunity of Galleria mellonella and Calliphora vicina
Source: Sci Rep. 2021 Aug 5;11:15963. doi: 10.1038/s41598-021-95440-6 (PMC8342708; doi:10.1038/s41598-021-95440-6)
Supplement: Supplementary file 1 — Supplementary Table 1. [file 41598_2021_95440_MOESM1_ESM.docx]

**Dodecanol, metabolite of entomopathogenic fungus Conidiobolus coronatus, affects fatty acid composition and cellular immunity of Galleria mellonella and Calliphora vicina**

Michalina Kazek^1*^, Agata Kaczmarek^1^, Anna Katarzyna Wrońska^1^, Mieczysława Irena Boguś ^1,2^

^1^The Witold Stefański Institute of Parasitology, Polish Academy of Sciences, 00-818 Warszawa, ul. Twarda 51/55, Poland

^2^BIOMIBO, 04-872 Warszawa ul. Strzygłowska 15, Poland

Correspondig author

* [m.kamut@twarda.pan.pl](mailto:m.kamut@twarda.pan.pl); +48226978973

**Supplementary Table 1.** Concentration of dodecanol (μg/ml±SD) determined by GC-MS in cell-free filtrates of *Conidiobolus coronatus.*

|  | MM medium 3w | LB medium 3w | MM medium 4w | LB medium 4w |
| --- | --- | --- | --- | --- |
| **DODECANOL** | 0.20±0.01^AC^ | 0.10±0.004^A^ | 0.25±0.01^BC^ | 0.18±0.02 |

**3w** – three weeks incubation; **4w –** four weeks incubation

Statistically significant differences are marked with the same letters (Student’s t-test, P≤0.05).

C. coronatus cultured in minimal medium (MM) produced significantly more dodecanol than the fungus propagated in rich (LB) medium.
