# Supplementary material for: Dodecanol, metabolite of entomopathogenic fungus Conidiobolus coronatus, affects fatty acid composition and cellular immunity of Galleria mellonella and Calliphora vicina
Source: Sci Rep. 2021 Aug 5;11:15963. doi: 10.1038/s41598-021-95440-6 (PMC8342708; doi:10.1038/s41598-021-95440-6)
Supplement: Supplementary file 2 — Supplementary Table 2. [file 41598_2021_95440_MOESM2_ESM.docx]

**Dodecanol, metabolite of entomopathogenic fungus *Conidiobolus coronatus*, affects fatty acid composition and cellular immunity of *Galleria mellonella* and *Calliphora vicina***

Michalina Kazek^1*^, Agata Kaczmarek^1^, Anna Katarzyna Wrońska^1^, Mieczysława Irena Boguś ^1,2^

^1^The Witold Stefański Institute of Parasitology, Polish Academy of Sciences, 00-818 Warszawa, ul. Twarda 51/55, Poland

^2^BIOMIBO, 04-872 Warszawa ul. Strzygłowska 15, Poland

Correspondig author

* [m.kamut@twarda.pan.pl](mailto:m.kamut@twarda.pan.pl); +48226978973

**Supplementary Table 2.** Total list of all cuticular free fatty acids (FFA) identified on the cuticle of *Galleria mellonella* and *Calliphora vicina* using GC/MS, in both larvae and adults (untreated insects or those treated with only the dodenaol solvent: acetone or ethanol).

| **Nr** | **Symbol** | **FFA** | **Semi-structural formula** | ***Galleria mellonella*** | | ***Calliphora vicina*** | |
| --- | --- | --- | --- | --- | --- | --- | --- |
|  |  |  |  | **Larvae** | **Adults** | **Larvae** | **Adults** |
| **1** | **C 4:0** | **Butanoic acid** | CH_3_(CH_2_)_2_COOH | **+** | **-** | **-** | **-** |
| **2** | **C 5:0** | **Pentanoic acid** | CH_3_(CH_2_)_3_COOH | **+** | **-** | **-** | **-** |
| **3** | **C 6:0** | **Hexanoic acid** | CH_3_(CH_2_)_4_COOH | **+** | **+** | **-** | **+** |
| **4** | **C 7:0** | **Heptanoic acid** | CH_3_(CH_2_)_5_COOH | **+** | **+** | **-** | **-** |
| **5** | **C 8:0** | **Octanoic acid** | CH_3_(CH_2_)_6_COOH | **+** | **+** | **-** | **+** |
| **6** | **C 9:0** | **Nonanoic acid** | CH_3_(CH_2_)_7_COOH | **+** | **+** | **-** | **+** |
| **7** | **C 10:0** | **Decanoic acid** | CH_3_(CH_2_)_8_COOH | **+** | **+** | **-** | **+** |
| **8** | **C 11:0** | [**Undecanoic**](https://pl.wikipedia.org/w/index.php?title=Kwas_undekanowy&action=edit&redlink=1) **acid** | CH_3_(CH_2_)_9_COOH | **-** | **+** | **-** | **-** |
| **9** | **C 12:0** | **Dodecanoic acid** | CH_3_(CH_2_)_10_COOH | **-** | **+** | **+** | **-** |
| **10** | **C 13:0** | [**Tridecanoic**](https://pl.wikipedia.org/w/index.php?title=Kwas_tridekanowy&action=edit&redlink=1) **acid** | CH_3_(CH_2_)_11_COOH | **-** | **+** | **-** | **-** |
| **11** | **C 14:1** | **Tetradecenoic acid** | CH_3_(CH_2_)_3_CH= =CH(CH_2_)_7_COOH | **+** | **+** | **+** | **+** |
| **12** | **C 14:0** | **Tetradecanoic acid** | CH_3_(CH_2_)_12_COOH | **+** | **+** | **+** | **+** |
| **13** | **C 15:0** | [**Pentadecanoic**](https://pl.wikipedia.org/w/index.php?title=Kwas_pentadekanowy&action=edit&redlink=1) **acid** | CH_3_(CH_2_)_13_COOH | **+** | **+** | **+** | **+** |
| **14** | **C 16:1** | **Hexadecenoic acid** | CH_3_(CH_2_)_5_CH= =CH(CH_2_)_7_COOH | **+** | **+** | **+** | **+** |
| **15** | **C 16:0** | **Hexadecanoic acid** | CH_3_(CH_2_)_14_COOH | **+** | **+** | **+** | **+** |
| **16** | **C 17:1** | **Heptadecenoic acid** | CH_3_(CH_2_)_6_CH= =CH(CH_2_)_7_COOH | **-** | **-** | **+** | **+** |
| **17** | **C 17:0** | **Heptadecanoic acid** | CH_3_(CH_2_)_15_COOH | **-** | **-** | **+** | **+** |
| **18** | **C 18:2** | **Octadecadienic acid** | CH_3_(CH_2_)_3_(CH_2_CH= =CH)_2_(CH_2_)_7_COOH | **-** | **+** | **-** | **+** |
| **19** | **C 18:1** | **Octadecenoic acid** | CH_3_(CH_2_)_7_CH= =CH(CH_2_)_7_COOH | **+** | **+** | **+** | **+** |
| **20** | **C 18:0** | **Octadecanoic acid** | CH_3_(CH_2_)_16_COOH | **+** | **+** | **+** | **+** |
| **21** | **C 20:1** | **Eicosenoic acid** | CH_3_(CH_2_)_7_CH= =CH(CH_2_)_9_COOH | **+** | **+** | **-** | **-** |
| **22** | **C 20:0** | **Eicosanoic acid** | CH_3_(CH_2_)_18_COOH | **-** | **-** | **-** | **+** |
| **23** | **C 22:0** | **Docosanoic acid** | CH_3_(CH_2_)_20_COOH | **-** | **-** | **-** | **+** |
| **24** | **C 24:0** | **Tetracosanoic acid** | CH_3_(CH_2_)_22_COOH | **-** | **-** | **-** | **+** |
| **25** | **C 26:0** | **Hexacosanoic acid** | CH_3_(CH_2_)_24_COOH | **-** | **-** | **-** | **+** |

**+** FFA occurred; **–** no occurrence

**ACN** – acetone control; **EtOH** – ethanol control
